# Single-cell landscape of immunological responses in patients with juvenile idiopathic arthritis

**DOI:** 10.1016/j.gendis.2025.101577

**Published:** 2025-03-03

**Authors:** Yun Liu, Xiwen Luo, Liuqing Yang, Qiang Luo, Xiya Luo, Li Xu, Yating Wang, Yunfei An, Yupeng Cun, Xuemei Tang

**Affiliations:** a Department of Rheumatology and Immunology, Children’s Hospital of Chongqing Medical University, Chongqing 400014, China; b Chongqing Key Laboratory of Child Rare Diseases in Infection and Immunity, National Clinical Research Center for Child Health and Disorders, Ministry of Education Key Laboratory of Child Development and Disorders, Children’s Hospital of Chongqing Medical University, Chongqing 400014, China; c Chongqing Key Laboratory of Pediatrics, National Clinical Research Center for Child Health and Disorders, Ministry of Education Key Laboratory of Child Development and Disorders, Children’s Hospital of Chongqing Medical University, Chongqing 400014, China

**Keywords:** Cell-cell communication, Immune cell, Juvenile idiopathic arthritis, Pseudotimetrajectories, Single-cell RNA sequencing

## Abstract

The study aimed to analyze the single-cell transcriptomes of immune cells in juvenile idiopathic arthritis (JIA) patients to understand the cellular heterogeneity within the immune system. Peripheral blood samples from fourteen JIA patients and four healthy individuals were subjected to single-cell RNA sequencing. Various subtypes of JIA were included in the patient cohort. Functional analyses, such as pseudotime trajectories and cell communication studies, were conducted to uncover immune cell changes in JIA patients. Results showed disrupted interferon and acute inflammatory responses in most cell types of JIA patients, with particularly intense responses in systemic JIA (sJIA) patients versus non-sJIA patients. Pseudotime analysis of CD4^+^ T, CD8^+^ T, B, and myeloid cells revealed that the functions of each cytokine production, cytotoxicity, and the processing and presentation of antigens were progressively strengthened, while the regulation of nuclear factor kappa B (NF-κB)-related pathways was weaker in CD4^+^ T and CD8^+^ T cells than in non-JIA. Reclustering analysis of myeloid cells highlighted interferon-related functions predominantly in non-classical monocytes of sJIA patients. Additionally, cell communication analysis identified unique ligand–receptor pairs in sJIA, suggesting potential roles in disease progression. In conclusion, interferon disorders are evident across various immune cell types in JIA patients, with stronger responses observed in sJIA patients. The ligand–receptor pairs involving migration inhibitory factor (MIF) and CXCR7/CD44 may contribute to differing joint symptoms between sJIA and non-sJIA patients. Moreover, non-classical monocytes and the CXCR2 receptor in MIF signaling may play crucial roles in sJIA progression.

## Introduction

Juvenile idiopathic arthritis (JIA) is not a distinct ailment but rather a term encompassing several types of arthritis of unknown origin. It persists for a duration of ≥6 weeks and typically emerges before the age of 16.^1^^,^^2^ In the late 1990s, the International League Against Rheumatism (ILAR) classified JIA into seven types according to the signs and symptoms seen in the first six months of the disorder.^3^^,^^4^ The categories were systemic arthritis (sJIA), rheumatoid factor-negative polyarthritis (RF^−^ pJIA), rheumatoid factor-positive polyarthritis (RF^+^ pJIA), oligoarthritis (oJIA), and enthesitis-related arthritis (ERA), as well as psoriatic and undifferentiated arthritis. Given the rarity of the last two subtypes in clinical practice, our study collectively refers to RF^−^ pJIA, RF^+^ pJIA, oJIA, and ERA as non-sJIA.

Apart from systemic JIA (sJIA), most studies on the genetics and immunology of the disease have focused on the broad JIA population. Comprehensive analyses of studies on the pathogenesis of JIA have shown that, despite differences, it is possible to group most of the JIA categories, with sJIA being the notable exception.^5^^,^^6^ This holistic approach emphasizes features, particularly in pathways, common to the different JIA types, through the investigation of interaction networks rather than examining isolated immune factors. Although the pathophysiology of JIA is not fully understood, it appears that disruption of the equilibrium between regulatory and effector immune cells induced by both genetic and environmental factors is involved.

It appears that the induction and persistence of JIA are primarily driven by elements within the adaptive immune system, pointing to the significance of consistent and chronic immune responses. Among these components, effector T cells are closely involved in JIA pathogenesis. Indications of potentially pathogenic antigens include the oligoclonality observed in specific T cell receptor subsets and in an elevated inflammatory response, often linked to the activity of the disease. These responses are fuelled by a diverse array of antigens originating from various sources, sharing availability, and potentially experiencing overexpression within inflamed microenvironments.^7^, ^8^, ^9^, ^10^, ^11^, ^12^

Accumulating evidence indicates the metabolic resilience and immune suppression resistance of effector T cell function, contributing to a self-reverberating and amplifying immune process. T cell subsets with similar T cell receptor repertoires, degrees of activation, and functional and immunological phenotypes, are observed both in the inflamed synovia and peripheries of individuals with active disease. These subsets, irrespective of their similarities, have both regulatory and effector properties. This duality suggests a certain plasticity in the function that may be influenced by microenvironmental factors and suggests that an understanding of the processes involved may be crucial for unraveling the precise pathogenetic mechanisms and optimizing therapeutic interventions.^12^, ^13^, ^14^, ^15^

B cells, together with T cells, are closely involved in adaptive immune functions contributing to the pathogenesis of JIA. B cells are known to generate autoantibodies, including antinuclear antibodies and rheumatoid factors, both of which are significant in the diagnosis of JIA.^16^^,^^17^ Despite numerous hypotheses and extensive research, there is no conclusive evidence to establish the primary involvement of autoantibodies in JIA pathogenesis. Notably, B cells are associated with the presentation of lipid and peptide antigens, inducing both pro- and anti-inflammatory cytokines. Emphasizing these characteristics is crucial, as they may contribute to the enhancement of aberrant immune function in JIA.^17^ B cells, in contrast to dendritic cells, lack the array of costimulatory molecules crucial for modulating T cell responses, thus potentially leading to abnormal activation of T cells. Additionally, B cells can produce chemokines and cytokines directly influencing the inflammatory process. Collectively, these immune cells appear to be closely linked with JIA pathogenesis and may thus offer novel therapeutic targets.^16^

The role of innate immunity is paramount in mediating and sustaining autoimmune damage.^18^^,^^19^ High-dimensional approaches have revealed notable abnormalities relative to controls in the functions and phenotypes of different types of innate immune cells, such as dendritic cells, natural killer cells, neutrophils, and macrophages.^20^^,^^21^ Many of these cells, such as neutrophils, tend to gather at sites of inflammation, possibly exacerbating the autoimmune response. Furthermore, microRNAs and acellular substances have been observed to contribute to JIA pathophysiology.^20^^,^^21^

To date, no comprehensive single-cell sequencing studies of the JIA subtypes have been performed. Here, single-cell RNA sequencing was used to comprehensively evaluate immune activity in peripheral blood mononuclear cells from patients diagnosed with primary JIA. The findings of this high-resolution, single-cell, transcriptomic analysis will enhance the understanding of the immune response, both pathogenic and protective, in JIA progression.

## Methods and materials

### Patient cohort

Three cases were obtained from the Gene Expression Omnibus (GEO) database, specifically GSE168732. The study cohort comprised fourteen patients diagnosed with JIA, alongside a single individual serving as a healthy control (HC). The recruitment of participants took place at the Children’s Hospital of Chongqing Medical University. The participants were classified into five clinical groups, namely, patients with sJIA, RF^−^ pJIA, RF^+^ pJIA, oJIA, and ERA, as defined by the ILAR criteria. Ethical approval of the protocol was granted by the Ethics Committee of the Children’s Hospital of Chongqing Medical University (file number: 2022 No. 52). Prior to sample collection, informed consent was provided by both pediatric patients and their legal guardians.

### Single-cell suspension preparation and single-cell RNA sequencing

Peripheral blood mononuclear cells were speedily isolated from 14 patients diagnosed with primary JIA and one control within 6 h of venous blood extraction. The cell viability surpassed 90% in each sample. The cells were cryopreserved at −80 °C until further use.

Cell sorting was performed in phosphate buffer saline with 0.05% bovine serum albumin according to the 10 × Genomics protocol. The time interval between preparation and loading on the 10 × Chromium controller was less than 2 h. Cells were counted and their viability was assessed microscopically with trypan blue, with viability >85% used as the criterion for sequencing.

Library construction was undertaken with the Single Cell 3’ Library Kit V2 from 10 × Genomics. Droplet sequencing was used to obtain the transcriptomic profiles of the cells, according to the 10 × Genomics protocol. The cDNA libraries were sequenced with paired-end reads, on an Illumina NovaSeq 6000 system.

### Single-cell RNA sequencing data processing

Raw expression matrices were produced for all samples using CellRanger (v.7.2.0) with the human genome assembly GRCh38. After output filtering, the matrices were analyzed using the Seurat 4.3 package (v.4.3.0) in R (v.4.3.0). Genes expressed in a proportion of over 0.1% of the data and cells with more than 300 genes were selected for further analyses.

After excluding poor-quality cells, the expression matrices were normalized using the function NormalizeData. The FindVariableFeatures function was used to determine 2000 features with high levels of cell-to-cell variation and the RunPCA function with default parameters was used for reducing the dataset dimensionalities after the generation of linear-transformed scaled data using the function ScaleData.

Additional functions, namely, ElbowPlot, JackStrawPlot, and DimHeatmap, were used for determining the true dimensionalities of the datasets, in accordance with the Seurat guidelines. Lastly, cell clustering was done using the FindClusters and FindNeighbors functions, and the function RunUMAP with default parameters was used for nonlinear dimensional reduction.

### Multiple dataset integration

For the comparative analysis of the cells present and their proportions in the samples, the integration procedures provided in the Seurat documentation at https://satijalab.org/seurat/v3.0/integration.html44 were used. Specifically, different datasets were integrated using the Seurat package (v.4.3.0) to create an unbatched and integrated dataset.

In a concise summary of the procedure, 2000 features that varied significantly between cells were identified, as described above. Subsequently, “anchors” between the specific datasets were pinpointed using the function FindIntegrationAnchors. The anchors were then used to produce a batch-corrected expression matrix for all cells using the function IntegrateData. This integration step facilitated the seamless combination and analysis of cells from different datasets.

### Annotation of cell types and identification of cluster markers

After reducing the nonlinear dimensions and the creation of a two-dimensional projection of the cells using UMAP, clustering of the cells was performed according to shared characteristics. Markers for the clusters were identified using the function FindAllMarkers. The clusters were annotated and categorized according to the presence of markers linked to specific cell types. To maintain the integrity of the analysis, clusters with two or more markers were classified as double cells and were not used for further analysis.

### Identification of DEGs and their functional analysis

Differentially expressed genes (DEGs) were identified with the function FindMarkers, using the default “Wilcox” test. The false discovery rate was assessed using the Benjamini-Hochberg method and filtering of DEGs was performed using the criteria log2(fold change) ≥ 0.5 and the false discovery rate <0.01. The functional enrichments of the DEGs were investigated using Metascape (www.metascape.org) with the gene ontology (GO) biological process category.

### Cell scores

Cell scores were employed to assess the extent of expression of specific gene sets by individual cells.^20^, ^21^, ^22^, ^23^ The initial calculation of the scores was determined according to the average expression of genes in a predefined set of individual cells. The control gene set represented a random selection according to aggregate expression level bins, such that the levels and oversizes were similarly distributed to the gene set under consideration. The implementation of this method, with default settings, was carried out using the AddModuleScore function in Seurat. We specifically utilized gene sets for “Response to interferon (IFN)-α” (GO:0035455), “Response to IFN-β” (GO:0035456), “Acute inflammatory response” (GO:0002526), and “B cell proliferation” (GO:0042100) to evaluate the IFN-α/β response, inflammatory responses, and B cell proliferation, respectively.

### Pseudotme analysis of epithelial cells and cell-chat signaling network analysis

Pseudo-timing analysis of CD4^+^ T, CD8^+^ T, and B cells, as well as myeloid cell subsets, was conducted using default parameters in monocle3 software. The developmental trajectories of the cells were inferred and the cells were ordered according to transcriptomic changes. To assess potential intercellular communication between immune cells, we utilized the R package CellChat 1.6.1.^24^

### Statistics

The statistical methods, tools, and thresholds used for the analyses are provided in detail in the *Results*, *Methods*, or figure legends.

## Results

### Single-cell transcriptomic analyses of peripheral immune cells in JIA

Droplet-based single-cell RNA sequencing of peripheral blood mononuclear cells from 14 JIA patients and 4 HCs was performed on the 10X Genomics platform. The 14 JIA patients were further categorized into five clinical subtypes, namely, sJIA (*n* = 4), RF^−^ pJIA (*n* = 2), RF^+^ pJIA (*n* = 2), oJIA (*n* = 3), and ERA (*n* = 3) (Table S1). Employing a single-cell analysis pipeline (described in the *Methods*), we obtained 177,310 cells among all samples, 27,233 cells (15.6%) originated from HCs, 39,494 cells (22.3%) from sJIA, 21,013 cells (11.9%) from RF^−^ pJIA, 24,136 cells (13.6%) from RF^+^ pJIA, 32,604 cells (18.4%) from oJIA, and 32,830 cells (18.5%) from ERA. After the removal of poor-quality cells, an unbatched integrated dataset was obtained.

**Figure 1:**
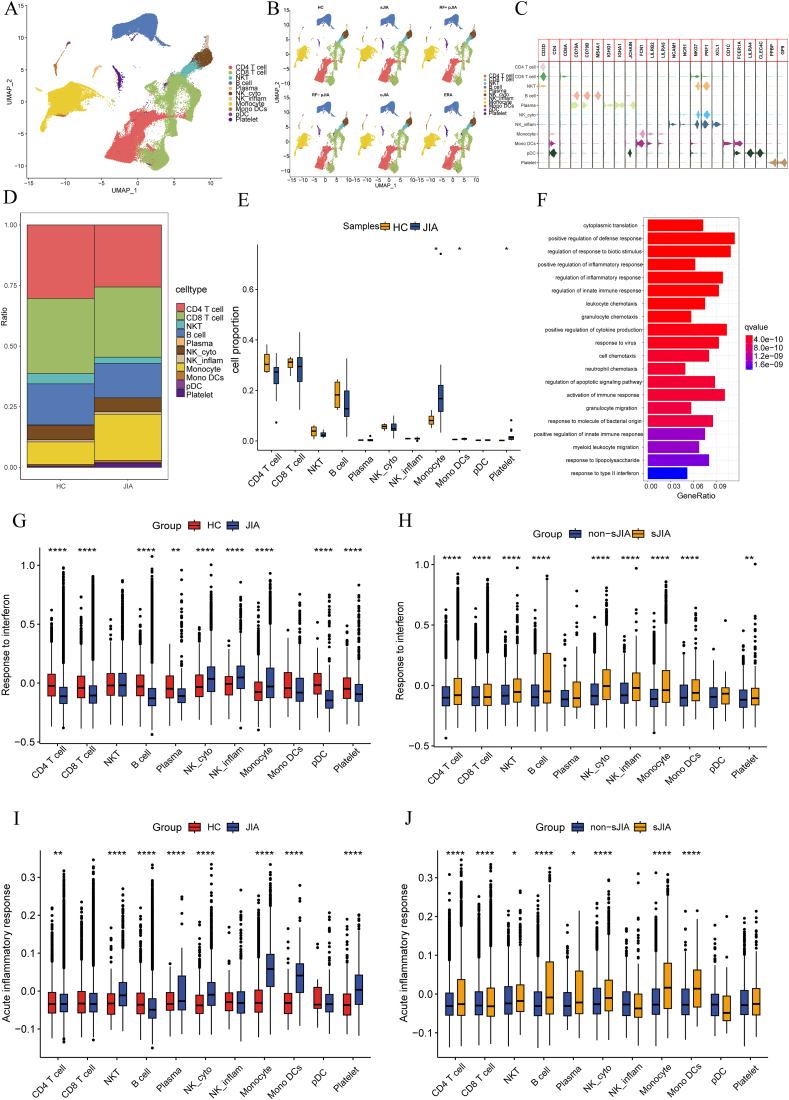
Single-cell transcriptomic profiles of PBMCs from JIA patients and HCs. **(A, B)** Identified cell populations. Eleven clusters were found after UMAP clustering of JIA and HC cells. The dots indicate single cells with colors representing the cell type. **(C)** Violin plots of expression of cell markers. **(D)** Relative proportions of cell subtypes in JIA and HC PBMCs. **(E)** Percentage of each cellular population in HC and JIA. **(F)** Enrichment analysis of HC and JIA for DEGs in monocyte. **(G**–**J)** The box plots of the levels of two gene ontology biological process terms in eleven clusters from HC, JIA, non-sJIA, and sJIA samples. PBMCs, peripheral blood mononuclear cells; JIA, juvenile idiopathic arthritis; sJIA, systemic juvenile idiopathic arthritis; HC, healthy control; DEGs, differentially expressed genes.

After reducing dimensionality, the cells were clustered and marker genes' characteristics of 10 principal cell types/subtypes were assigned (Fig. 1A). These cell types encompassed CD4^+^ (CD3D^+^CD4^+^), CD8^+^ (CD3D^+^CD8A^+^CD8B^+^), and natural killer (NK) (CD3D^+^NKG7^+^PRF1^+^) T cells, as well as B (CD79A^+^CD79B^+^MS4A1^+^), plasma (IGHG1^+^IGHA1^+^JCHAIN^+^), NK cytotoxicity (NKG7^+^PRF1^+^), NK inflammation (NKG7^+^PRF1^+^XCL1^+^) cells, monocytes (FCN1^+^LILRB2^+^LILRA5^+^), monocyte-derived dendritic cells (CD1C^+^), plasmacytoid dendritic cells (LILRA4^+^), and platelets (PPBP^+^) (Fig. 1B). This provided a clear delineation of the present cell populations.

To elucidate disparities in cellular compositions at two levels, JIA versus HC and non-sJIA versus sJIA, the relative proportions of the 10 principal cell types identified in peripheral blood mononuclear cells were determined according to the single-cell RNA sequencing data (Fig. 1C). At the JIA versus HC level, three cell populations, monocytes, monocyte-derived dendritic cells, and platelets, exhibited significant elevation in JIA (Fig. 1D). Next, to explore the immune response in JIA patients, enrichment in two GO biological process pathways, namely, response to IFN-α and acute inflammatory response, were assessed in the cell types across the four conditions (Fig. 1E), as the GO enrichment analyses of DEGs in various subpopulations of JIA and HC showed that some of the subpopulations' enrichment results were involved in IFN signaling (Fig. S1). We observed a uniform and significant up-regulation in response to IFN-α across most major cell types from the peripheral blood of JIA patients, except for monocytes and NK cells. Moreover, sJIA patients exhibited notably heightened responses to IFN-α in nearly all major cell types compared with non-sJIA (Fig. 1F). Furthermore, within the selected cell types, the acute inflammatory response differed significantly and consistently between the conditions. In comparison to HC, several cell types in JIA demonstrated significantly heightened performance in this process, with sJIA also exhibiting a significant up-regulation compared with non-sJIA. These findings indicate a more pronounced pro-inflammatory response in JIA patients, particularly in sJIA versus non-sJIA (Fig. 1G).

In conclusion, for most immune cells in JIA, IFN disorganization could be canalized and myeloid cells differed markedly between HC and JIA. There were different IFN disorders on most immune cells in the JIA.

### Characteristics of CD4^+^ T cell subsets in JIA

**Figure 2:**
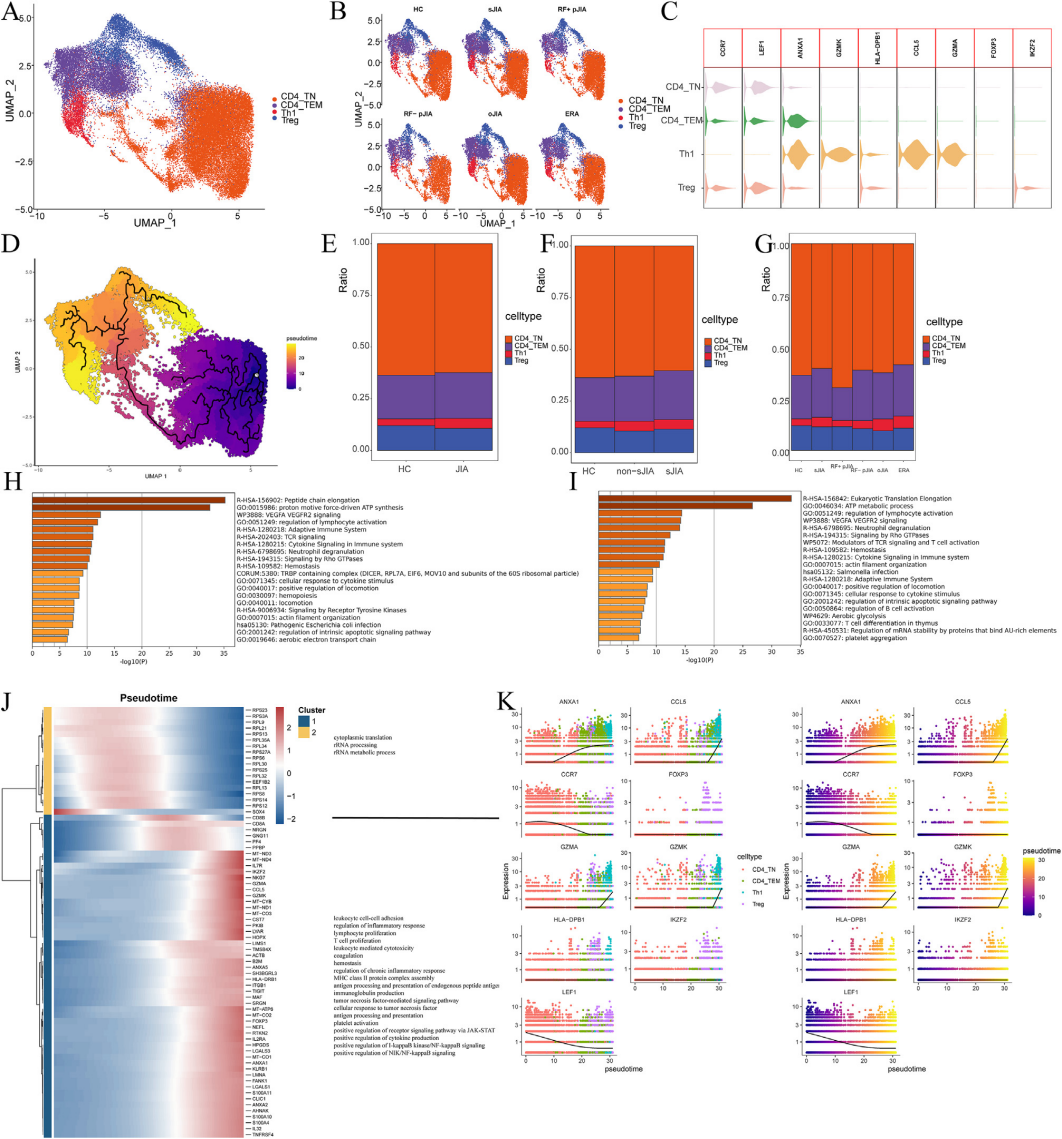
CD4^+^ T cell subtypes in JIA and HC PBMCs. **(A, B)** UMAP projection of CD4^+^ T cells. The dots represent single cells with colors representing the cell type. **(C)** Violin plots of distributions of cell marker expression in the four clusters. **(D)** Monocle3 assessment of pseudotime reconstructions and developmental trajectories of CD4^+^ T cells in PBMCs. **(E**–**G)** Relative proportions of cell subtypes in PBMCs from the different conditions. **(H, I)** DEG enrichment for CD4^+^ T between HC and non-sJIA (H) and between HC and sJIA (I). **(J)** Heatmap of changes in gene levels with pseudotime trajectories. **(K)** Changes in the expression of CD4^+^ T cell marker genes with pseudotime trajectories. PBMCs, peripheral blood mononuclear cells; JIA, juvenile idiopathic arthritis; sJIA, systemic juvenile idiopathic arthritis; HC, healthy control; DEGs, differentially expressed genes.

To elucidate alterations in these subsets, CD4^+^ T cells were subclustered from peripheral blood mononuclear cells, identifying five subsets according to the distributions and expression of defined CD4^+^ T cell markers (Fig. 2A, B): naive CD4^+^ (CD4^+^ TN; CCR7^+^LEF1^+^), effector memory CD4^+^ (CD4^+^ TEM; ANXA1^+^), and T-helper type 1 (Th1; HLA-DPB1^+^CCL5^+^GZMA^+^) T cells, as well as regulatory T cells (Tregs) (FOXP3^+^IKZF2^+^).

We then utilized Monocle3 to infer pseudotime trajectories of the CD4^+^ T cell subsets. We observed two distinct trajectories: CD4^+^ TN cells connected to CD4^+^ TEM cells, which subsequently differentiated into two distinct cell lines, Treg and Th1 (Fig. 2C). With the progression of cellular trajectories, the regulation of various cytokines, T cell proliferation, chronic inflammatory response regulation, MHC class II protein complex assembly, immunoglobulin production, tumor necrosis factor-mediated signaling pathway, cellular response to tumor necrosis factor, antigen processing and presentation, platelet activation, positive regulation of receptor signaling pathway via Janus kinase (JAK)-signal transducer and activator of transcription (STAT), positive regulation of cytokine production, and positive regulation of NF-κB signaling were enhanced in JIA, while the cytoplasmic translations, rRNA processing, and rRNA metabolism were weakened (Fig. 2E).

For further evaluation of transcriptomic alterations in CD4^+^ cells, CD4^+^ cell profiles were compared between sJIA, non-sJIA, and HC cells. The greatest enrichment of DEGs from the sJIA and non-sJIA groups was associated with the regulation of intrinsic apoptotic signaling and VEGFA/VEGFR2 signaling pathways.

### Delineation of CD8^+^ T cell subsets and transcriptomic changes in JIA

**Figure 3:**
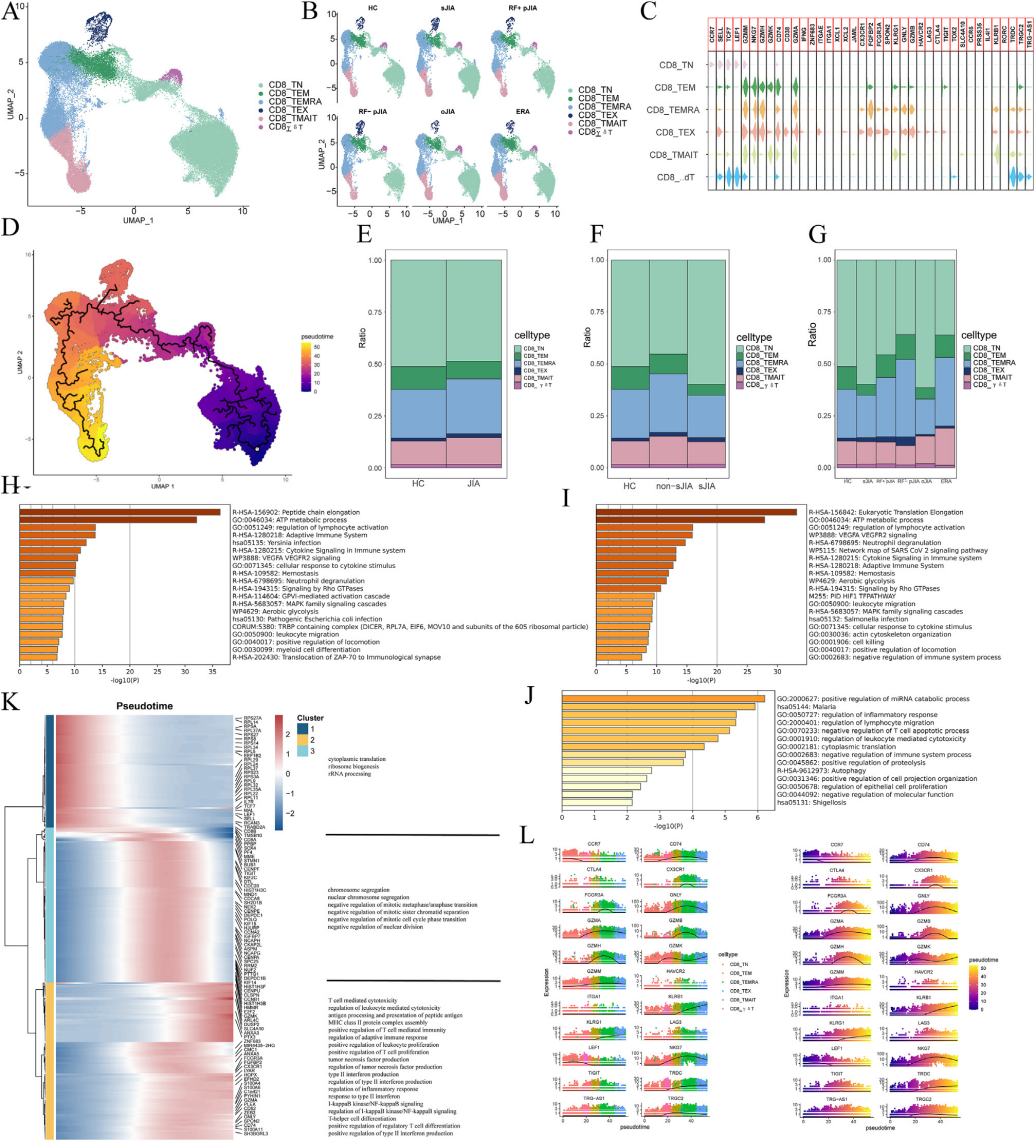
CD8^+^ T cell subtypes in JIA and HC PBMCs. **(A**, **B)** UMAP projection of CD4^+^ T cells. The dots represent single cells with colors representing the cell type. **(C)** Violin plots of the distributions of cell marker expression in the six clusters. **(D)** Monocle3 assessment of pseudotime reconstructions and developmental trajectories of CD8^+^ T cells in PBMCs. **(E**–**G)** Relative proportions of cell subtypes in PBMCs in each condition. **(H, I)** Enrichment of DEGs for CD8^+^ T cells was analyzed between HC and non-sJIA (H) and between HC and sJIA (I). **(J)** Heatmap of gene expression changes with pseudotime trajectories. **(K)** Expression changes of marker genes for CD4^+^ T cell with pseudotime trajectories. PBMCs, peripheral blood mononuclear cells; JIA, juvenile idiopathic arthritis; sJIA, systemic juvenile idiopathic arthritis; HC, healthy control; DEGs, differentially expressed genes.

To delineate changes in CD8^+^ T cell subsets, these cells were subclustered from peripheral blood mononuclear cells, leading to the identification of five subsets according to the presence of specific markers for these cells (Fig. 3A, B), namely, naive CD8^+^ (CD8^+^ TN; CCR7^+^TCF7^+^LEF1^+^), effector memory CD8^+^ (CD8^+^ TEM; GZMK^+^GZMM^+^), recently activated effector-memory (CD8^+^ TEMRA; CX3CR1^+^, FGFBP2^+^, FCGR3A^+^), exhaustion-like CD8^+^ (CD8^+^ TEX, with markers GZMA, GNLY, GZMB, and IFNG), mucosal-associated invariant (TMAIT; SLC4A10, PRSS35, CCR6), and gamma-delta (Tγδ; TRDC^+^, TRGC2^+^, and TRG-AS1^+^) T cells.

Furthermore, we extrapolated pseudotime trajectories. We observed three distinct trajectories: the first involved CD8^+^ TN and γδT cells, the second encompassed CD8^+^ TN, CD8^+^ TEM, and CD8^+^ TEX, and the third included CD8^+^ TN, CD8^+^ TEM, CD8^+^ TEMRA, and CD8^+^ TMAIT (Fig. 3C). As the cell trajectories progressed, functional changes in sJIA T cell-mediated cytotoxicity, MHC class II protein complex assembly, regulation of adaptive immune response, positive regulation of leukocyte proliferation, tumor necrosis factor production, type II IFN production, regulation of type II IFN production, regulation of inflammatory response, and regulation of NF-κB signaling were enhanced, whereas the cytoplasmic translations, rRNA processing, and ribosome biogenesis were weakened (Fig. 3E).

For further investigation of transcriptomic alterations in CD8^+^ cells, the profiles of CD8^+^ cells were compared between the sJIA, non-sJIA, and HC groups. DEGs in sJIA and non-sJIA patients with HC-involved genes related to VEGFA/VEGFR2 signaling. These DEGs of sJIA versus non-sJIA were significantly enriched in pathways associated with regulation of the inflammatory response, lymphocyte migration, leukocyte-mediated cytotoxicity, and negative regulation of T cell apoptotic processes. These results suggest that sJIA has a stronger inflammatory response, lymphocyte migration, and cytotoxicity than non-sJIA, and that apoptosis of T cells is stronger in non-sJIA.

### IFN production differs significantly between sJIA and non-sJIA B cells

**Figure 4:**
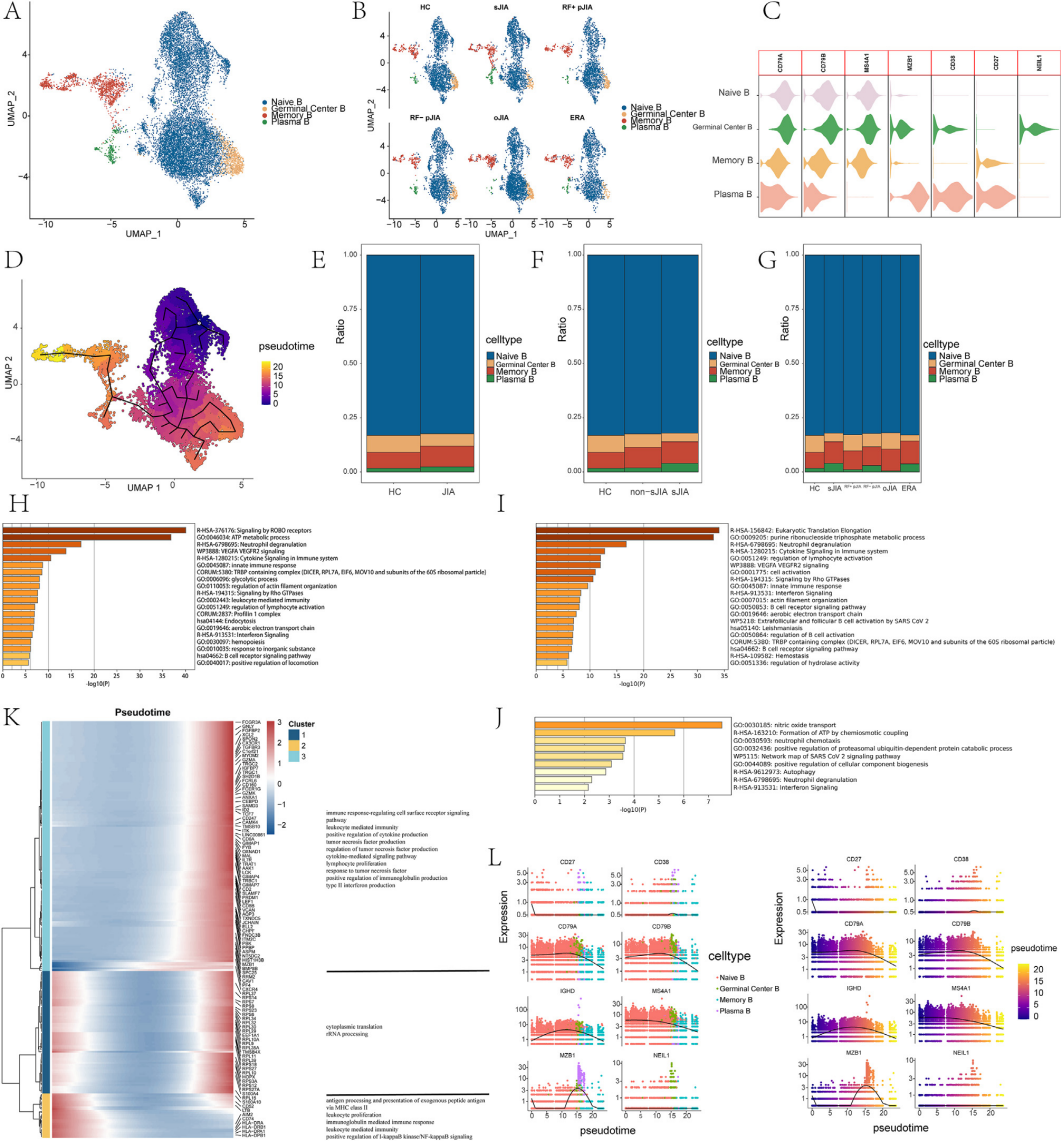
B cell phenotypes in JIA and HC PBMCs. **(A**, **B)** UMAP projection of CD4^+^ T cells. The dots represent single cells with colors representing cell types. **(C)** Violin plots of the distributions of expression of cell markers in the four clusters. **(D)** Monocle3 assessment of pseudotime reconstructions and developmental trajectories of CD8^+^ T cells in PBMCs. **(E**–**G)** Relative proportions of cell subtypes in PBMC in each condition. **(H**–**J)** Enrichment of DEGs for B cells was analyzed between HC and non-sJIA (H), between HC and sJIA (I), and between non-sJIA and sJIA (J). **(K)** Heatmap of gene expression changes with pseudotime trajectories. **(L)** Expression changes of marker genes for B cell with pseudotime trajectories. PBMCs, peripheral blood mononuclear cells; JIA, juvenile idiopathic arthritis; sJIA, systemic juvenile idiopathic arthritis; HC, healthy control; DEGs, differentially expressed genes.

To elucidate the changes in B cell subtypes, these cells were subclustered into four subtypes according to marker expression (Fig. 4A, B), namely, the naive B (MS4A1^+^IGHD^+^), memory B (MS4A1^+^CD27^+^), germinal center B (MS4A1^+^NEIL1^+^), and plasma B (MZB1^+^CD38^+^) subsets.

Cell proliferation scores were determined in the different subsets. In naive, germinal center, and memory B cell subsets, the scores were higher in non-sJIA versus sJIA (Fig. 4D). In the pseudotime trajectories (Fig. 4C), we observed immune response-regulating cell surface receptor signaling pathway, positive regulation of cytokine production, tumor necrosis factor production, cytokine-mediated signaling pathway, response to tumor necrosis factor, type II IFN production, and regulation of type II IFN production were enhanced, while antigen processing and presentation of exogenous peptide antigen via MHC class II, leukocyte proliferation, immunoglobulin-mediated immune responses, and regulation of NF-κB signaling were weakened (Fig. 4E), in contrast to those seen in CD4^+^ and CD8^+^ T cells.

DEGs in sJIA and non-sJIA were involved in genes related to VEGFA/VEGFR2 signaling and IFN signaling. DEGs of sJIA and non-SJIA patients were involved in IFN signaling, consistent with that the response to IFN was different in B cells and stronger in sJIA than in non-sJIA (Fig. 1). These results suggest that VEGFA/VEGFR2 signaling is linked to JIA pathogenesis to some degree and that IFN may be one of the factors that contribute to the different clinical manifestations of sJIA and non-sJIA.

### Non-classical monocytes are present only in sJIA

**Figure 5:**
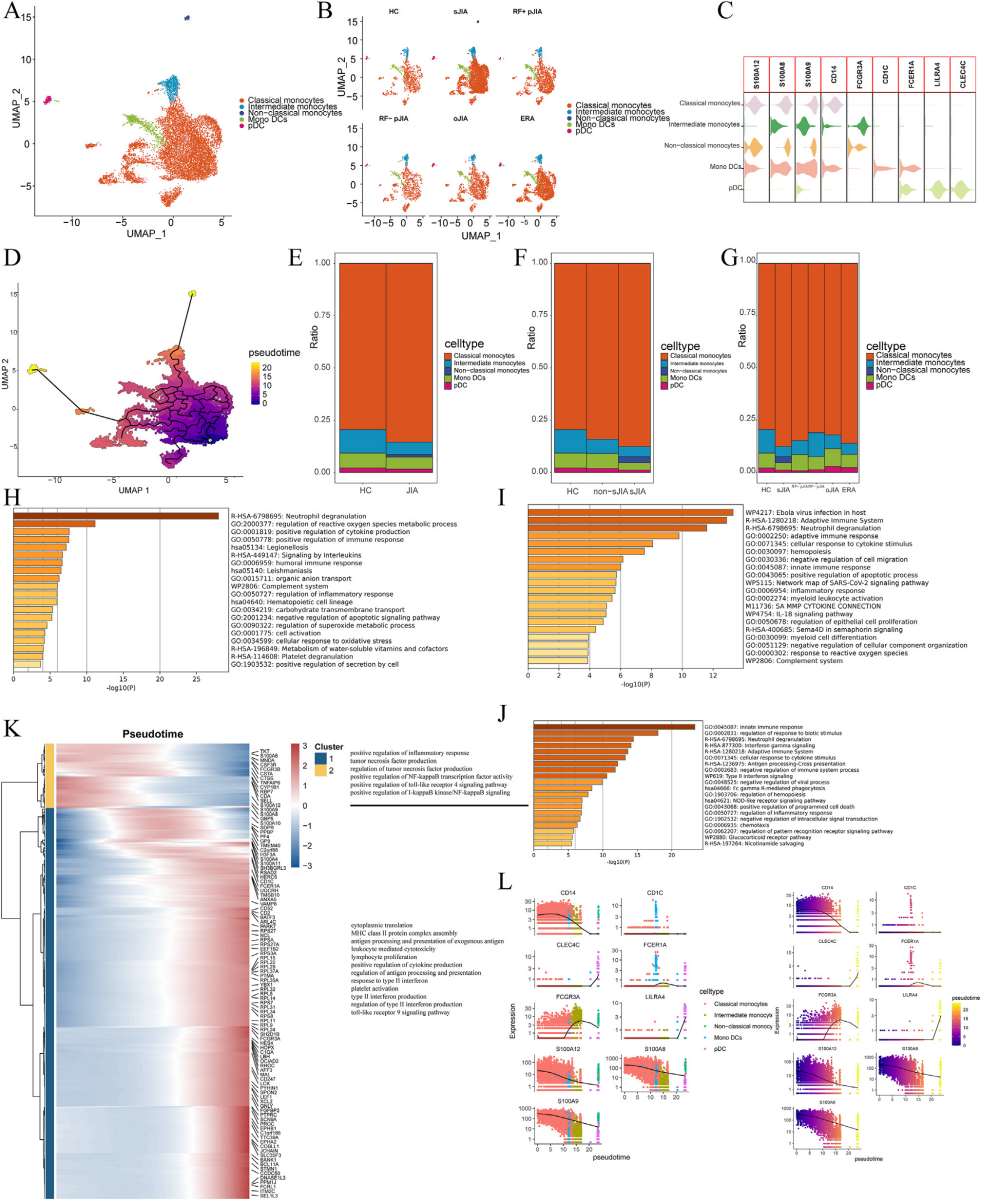
Myeloid cell phenotypes in JIA and HC PBMCs. **(A, B)** UMAP projection of CD4^+^ T cells. The dots represent single cells with colors representing cell types. **(C)** Violin plots of distributions of expression of cell markers in the four clusters. **(D)** Monocle3 assessment of pseudotime reconstructions and developmental trajectories of myeloid cells in PBMCs. **(E**–**G)** Relative proportions of cell subtypes in PBMCs in each condition. **(H**–**J)** Gene ontology enrichment analysis of highly variable genes for classical monocytes (H), intermediate monocytes (I), and non-classical monocytes (J). **(K)** Heatmap of gene expression changes with pseudotime trajectories. **(L)** Expression changes of marker genes for myeloid cells with pseudotime trajectories. PBMCs, peripheral blood mononuclear cells; JIA, juvenile idiopathic arthritis; HC, healthy control.

To delineate changes in myeloid cell subsets, these cells were subclustered from peripheral blood mononuclear cells and classified according to markers (Fig. 5A), namely, classical (CD14^+^FCGR3A^−^), intermediate (CD14^+^FCGR3A^+^), non-classical (CD14^−^FCGR3A^+^) monocytes, monocyte-derived dendritic cells (CD1C^+^FCER1A), and plasmacytoid dendritic cells (LILRA4^+^CLEC4C^+^). Interestingly, non-classical monocytes are a group of cells that are only found in sJIA (Fig. 5B). So, we did GO analysis of highly variable genes in each of the three groups of cells, namely, classical, intermediate, and non-classical monocytes.

We found that the top 20 entries in the classical monocytes were mainly enriched for positive regulation of cytokine production and the immune response, signaling by interleukins, humoral immune response, complementation system, regulation of inflammatory response, and platelet degranulation (Fig. 5E). Intermediate monocytes were mainly enriched in adaptive immune system, cellular response to cytokine stimuli, hemopoiesis, negative regulation of cell migration, innate immunity, positive regulation of apoptotic process, inflammatory response, myeloid leukocytes, platelet degranulation, inflammatory response, myeloid leukocyte activation, IL-18 signaling pathway, myeloid cell differentiation, and complement system, function like a response to the function of classical monocytes (Fig. 5F). Whereas non-classical monocytes were mainly enriched in the innate immune response, IFN-γ signaling, cellular response to cytokine stimulus, negative regulation of immune system process, type II IFN signaling, NOD-like receptor signaling pathway, positive regulation of programmed cell death, regulation of inflammatory response, and glucocorticoid receptor pathway (Fig. 5G), appeared to be associated with IFN, programmed cell death, and glucocorticoid receptor pathway-related functions.

In the pseudotime trajectories (Fig. 5C), we observed MHC class II protein complex assembly, antigen processing and presentation of exogenous antigen, leukocyte-mediated cytotoxicity, positive regulation of cytokine production, regulation of antigen processing and presentation, response to type II IFN, and type II IFN production were enhanced, while tumor necrosis factor production, regulation of tumor necrosis factor production, and positive regulation of NF-κB transcription factor activity were diminished (Fig. 5I). This implies that the production of IFN by non-classical monocytes might have a role in the generation of sJIA which needs to be further explored.

### Cell–cell interaction network analysis using CellChat

**Figure 6:**
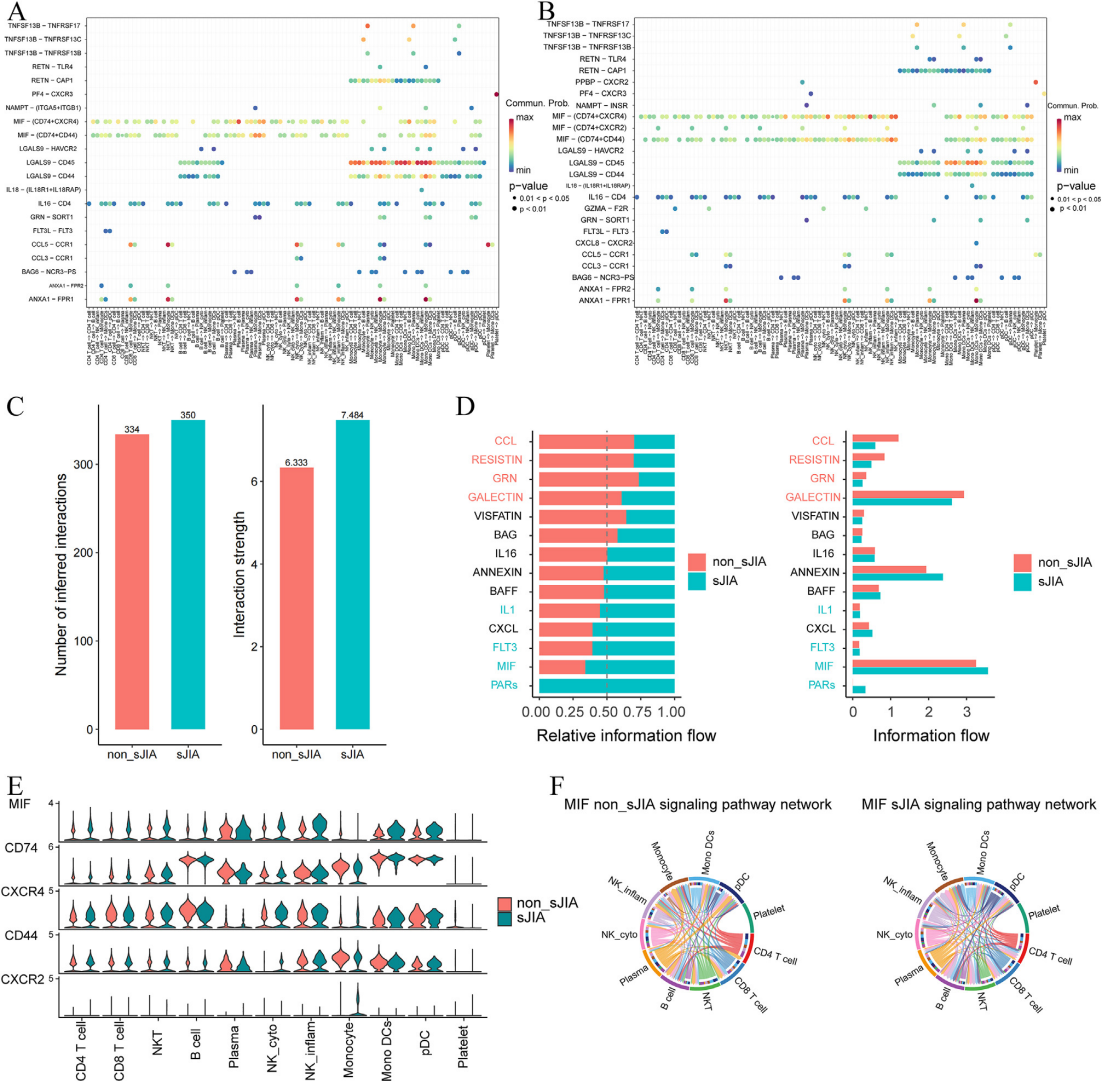
Cellular communication between immune cells. **(A, B)** Numbers of receptor-ligand interaction pairs predicted by CellChat in non-sJIA (A) and sJIA (B). **(C)** Numbers of predicted interactions and interaction strengths in non-sJIA and sJIA. **(D)** Information flow for all pathways and information flow in non-sJIA and sJIA. **(E)** Expression of all genes in MIF in non-sJIA and sJIA. **(F)** MIF signaling pathway network in immune cells in non-sJIA and sJIA. sJIA, systemic juvenile idiopathic arthritis; MIF, migration inhibitory factor.

CellChat was used for analysis of interactions between the identified cell types (Fig. S2). Notably, interactions of CD4^+^ and CD8^+^ T cells, B cells, monocytes, and NK cells with other cell populations were abundant in both sJIA and non-sJIA cases (Fig. 6A, B). Macrophage MIF was widely present in individual cells.

There is a greater number of inferred interactions in sJIA than in non-sJIA (Fig. 6C). In relative information flow, CCL, RESISTIN, GRN, and GALECTIN were mainly enriched in non-sJIA, and IL1, FLT3, MIF, and PARs were mainly enriched in sJIA (Fig. 6D). The CD74–CXCR4 and CD74–CD44 pairs were expressed in both sJIA and non-sJIA in MIF, but intriguingly, a new ligand–receptor pair, CD74–CXCR2, appeared in sJIA, whose ligand CXCR2 was expressed only in monocytes (Fig. 6E).

## Discussion

JIA is an arthritis occurring in children, and the sJIA and non-sJIA subtypes were analyzed in the present study. sJIA, in particular, stands out not only in clinical presentation but also in its pathogenesis, representing a potential polygenic autoinflammatory disease involving interconnections between innate and adaptive immunity.^25^ The early stages of the disease are marked by innate immunity-driven systemic inflammation. In contrast, non-sJIA is characterized by generalized joint involvement, with some patients facing an elevated risk of chronic iridocyclitis development. While studies have analyzed innate and adaptive immune responses individually, there is no comprehensive evaluation of the underlying molecular and cellular pathways involved in JIA. Here, a comprehensive single-cell analysis of JIA was performed, demonstrating the changes in cellular response associated with the progression of the disease and the identification of associated factors.

It was found that a significant portion of cellular responses to IFN exhibited a decrease. Conversely, in sJIA, IFN responses were heightened compared with non-sJIA. IFNs are pivotal immunomodulatory molecules orchestrating immune responses, combatting infections, and regulating inflammation. Individuals with JIA may display a diminished response to IFNs, potentially a consequence of immune system dysregulation. This diminished response might impair the immune system’s ability to effectively combat infections or inflammation, thereby exacerbating the condition in individuals with JIA. This phenomenon is likely intricately linked to the pathophysiological mechanisms of JIA, possibly arising from abnormal immune cell function, disrupted signaling pathways, or insufficient immune regulatory factors. sJIA, a specific subtype of JIA, is characterized by an immune system activation triggering a systemic inflammatory response. As the disease progresses, immune cells significantly amplify their response to IFNs. Consequently, an elevated response to IFNs is evident in patients with sJIA. This phenomenon is closely associated with the immune system’s activation state, the severity of inflammation in the disease, and the pathophysiology. It might signify a specific immune response to the disease, potentially modulating inflammation and immune responses to contend with disease progression.

IFN functions as a signaling protein synthesized and secreted by cells, belonging to three cytokine families.^26^ Upon specific binding to its cell membrane receptors, IFN activates the IFN signaling pathway, thereby inducing various IFN-stimulated genes. This activation is important in the modulation of the cellular immune system.^27^ A study concentrating on rheumatoid arthritis has discerned increased mRNA levels of IFN effectors in inflammatory cells, suggesting the involvement of IFN in the autoimmune response associated with rheumatoid arthritis. Notably, osteoclasts significantly contribute to irregular bone resorption, a phenomenon implicated in various bone diseases, including rheumatoid arthritis. Research indicates that Interferon-induced protein with tetratricopeptide repeats 1 (IFIT1) modulates receptor activator of nuclear factor κB ligand (RANKL) via STAT3 signaling, thereby facilitating osteoclast formation.^28^ Moreover, the observed increases in responses to IFN and acute inflammation in sJIA versus non-sJIA align with clinical characteristics. In the results of cellular communication, we observed that sJIA had one more ligand–receptor pair, MIF (CD74–CXCR2), than non-sJIA.

MIF acts as a pleiotropic pro-inflammatory cytokine involved in the modulation of both innate and adaptive immunity. It is associated with several autoimmune diseases, such as rheumatoid arthritis and systemic lupus erythematosus. Specifically, ligand–receptor pairs, such as MIF (CD74–CXCR4) and MIF (CD74–CD44), indicate the importance of CD44 in epithelia, cell proliferation, and certain cancers. The ectodomains of the protein promote migration through the extracellular matrix through interactions with growth factors, such as fibroblast growth factor, and matrix metalloproteinases.^29^ Cellular proliferation is important in the chronicity of arthritis, with activation and proliferation of B cells contributing to the progression of the disease to a chronic status. Here, greater B cell proliferation was seen in non-sJIA versus sJIA, corresponding to the increased severity of joint symptoms in these patients. Animal studies using collagen-induced arthritis as a rheumatoid arthritis model found that the severity of rheumatoid arthritis was reduced in the absence of MIF. This model causes inflammatory arthritis characterized by autoantibodies against collagen II driven largely by T cells. This aligns with our results and demonstrates the importance of MIF in these diseases.^30^, ^31^, ^32^ MIF has also been implicated in the pathogenesis of human systemic lupus erythematosus.^33^ The identification of the emerging ligand–receptor pair MIF (CD74–CXCR2) in sJIA may serve as an indicator to distinguish sJIA from non-sJIA.

In conclusion, this is the first characterization of the single-cell profiles of different subtypes of patients with primary JIA in a large sample size. We found that the myeloid cell population communicated more frequently with other cells in JIA than HC and that its subpopulation, non-classical monocytes, was present only in sJIA but not in non-sJIA. Moreover, the monocytes in sJIA showed a new ligand–receptor pair MIF (CD74–CXCR2) that was not observed in non-sJIA, and perhaps the expression of this receptor-ligand pair may help us to identify sJIA and non-sJIA.

Our findings hold significant implications for the precise diagnosis and classification of JIA, offering a foundation for future targeted therapies. By uncovering subtype-specific immune mechanisms, this study provides valuable insights that could guide the development of personalized treatment strategies for JIA patients. Although the number of cases in each subtype is relatively small, the high resolution and granularity of single-cell transcriptomics allow us to identify meaningful differences in immune cell populations and pathways between JIA subtypes. We are currently enrolling additional patients and performing detailed subtype analyses to enhance our understanding of JIA heterogeneity and optimize therapeutic strategies.

## CRediT authorship contribution statement

**Yun Liu:** Data curation, Methodology, Validation, Visualization, Writing – original draft. **Xiwen Luo:** Data curation, Investigation, Project administration, Writing – original draft. **Liuqing Yang:** Methodology, Visualization. **Qiang Luo:** Investigation, Methodology. **Xiya Luo:** Investigation, Methodology. **Li Xu:** Methodology. **Yating Wang:** Methodology. **Yunfei An:** Methodology. **Yupeng Cun:** Conceptualization, Methodology, Supervision, Validation, Visualization, Writing – review & editing. **Xuemei Tang:** Conceptualization, Data curation, Funding acquisition, Methodology, Project administration, Writing – review & editing.

## Data availability

All raw and processed data are available from the corresponding author upon reasonable request.

## Funding

This research was supported by the 10.13039/501100012166National Key Research and Development Program of China (No. 2021YFC2702003).

## Conflict of interests

The authors declared that the research was conducted in the absence of any commercial or financial relationships that could be construed as a potential conflict of interest.

## Appendix A Supplementary data

Multimedia component 1 Multimedia component 1

Multimedia component 2 **Figure S1** Single-cell transcriptomic profiles in PBMCs from JIA patients and HCs. **(A)** Clustering of cell types according to markers, shown by UMAP. Colors indicate expression levels. **(B–H)** Gene ontology enrichment analysis of DEGs in HC and JIA in CD4^+^ T cells (B), CD8^+^ T cells (C), B cells (D), monocytes (E), NK cytotoxicity cells (F), NK inflammation cells (G), and NK T cells (H). PBMCs, peripheral blood mononuclear cells; DEGs, differentially expressed genes; HC, healthy control; NK, natural killer. Multimedia component 2

Multimedia component 3 **Figure S2** Cellular communication between immune cells. **(A**–**D)** Several receptor-ligand pairs interacted between immune cells as predicted by CellChat in RF^+^ pJIA (A), RF^−^ pJIA (B), oJIA (C), and ERA (D). **(E, F)** Relative information flow for all pathways and information flow in RF^+^ pJIA (E) and RF^−^ pJIA (F). RF^+^ pJIA, rheumatoid factor-positive polyarthritis; RF^−^ pJIA, rheumatoid factor-negative polyarthritis; oJIA, oligoarthritis; ERA enthesitis-related arthritis. Multimedia component 3
