# Supplementary material for: Single-cell landscape of immunological responses in patients with juvenile idiopathic arthritis
Source: Genes Dis. 2025 Mar 3;12(5):101577. doi: 10.1016/j.gendis.2025.101577 (PMC12221592; doi:10.1016/j.gendis.2025.101577)
Supplement: Multimedia component 1 [file mmc1.pdf]

Supplementary Table 1. Clinical features of recruited donors.

Sample	Sex(male/female)	Age(years)	Country
H1	M	1.7	China
H2	F	3	China
H3	F	5	China
H4	M	12.4	China
sJIA1	F	6	China
sJIA2	M	6.5	China
sJIA 3	F	8.7	China
sJIA 4	F	11	China
RF+ pJIA1	F	2.5	China
RF+ pJIA2	F	8	China
RF- pJIA1	F	6.8	China
RF- pJIA2	M	7	China
oJIA1	F	9.4	China
oJIA2	M	10	China
oJIA3	M	13	China
ERA1	M	10.4	China
ERA2	M	10.8	China
ERA3	M	11.3	China
