# Supplementary figures and images for: Single-cell landscape of immunological responses in patients with juvenile idiopathic arthritis
Source: Genes Dis. 2025 Mar 3;12(5):101577. doi: 10.1016/j.gendis.2025.101577 (PMC12221592; doi:10.1016/j.gendis.2025.101577)

A

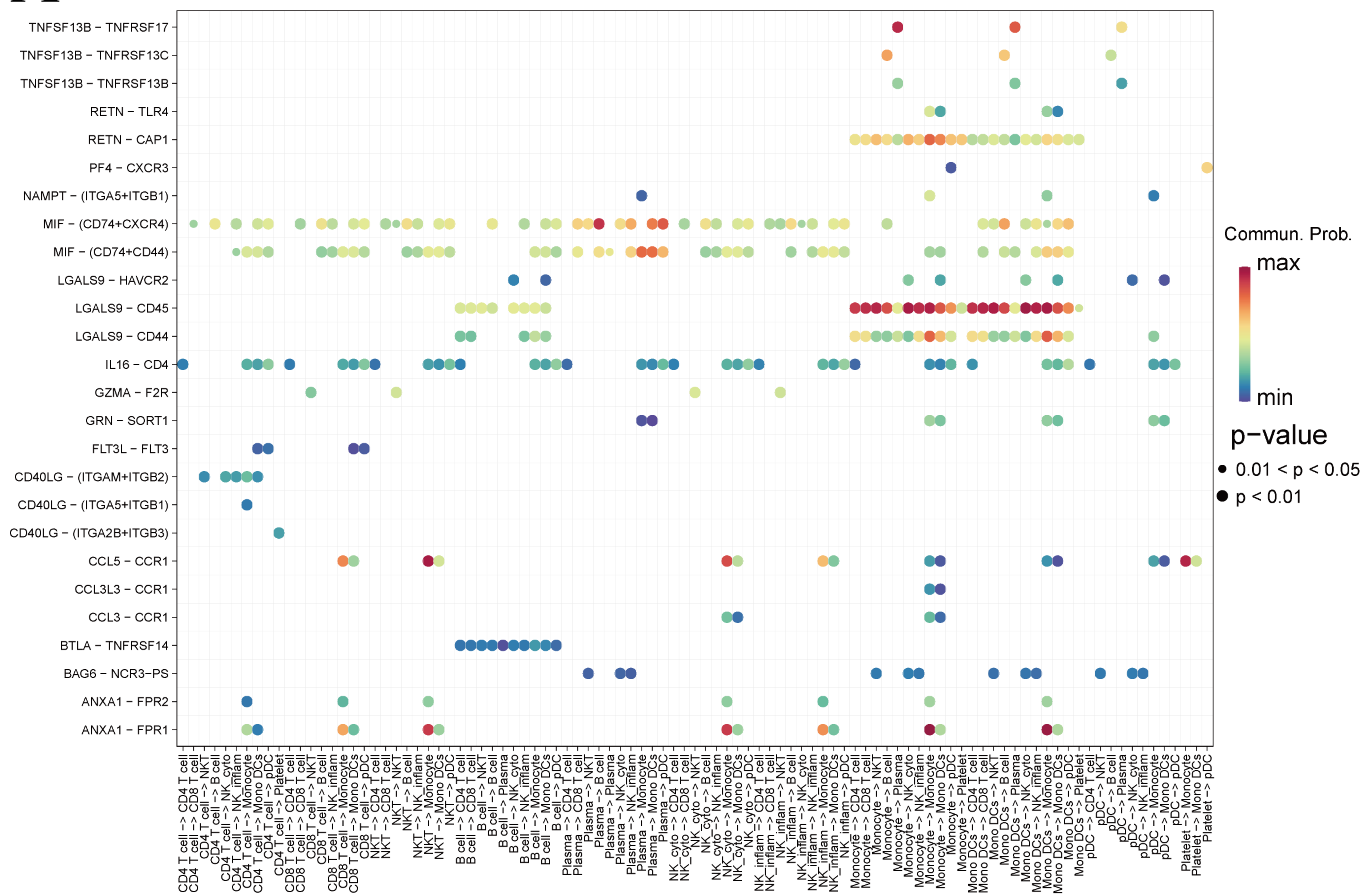

B

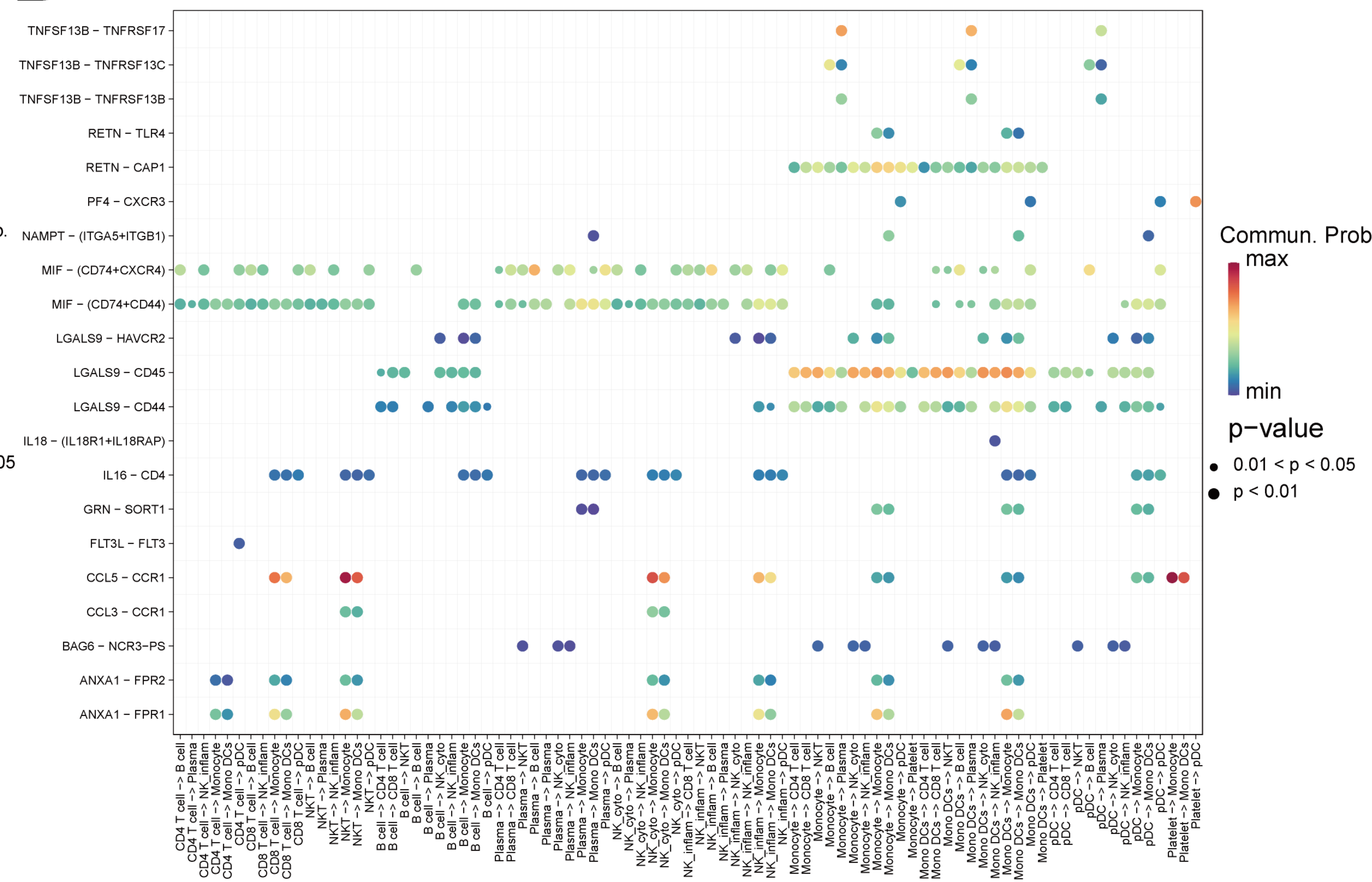

C

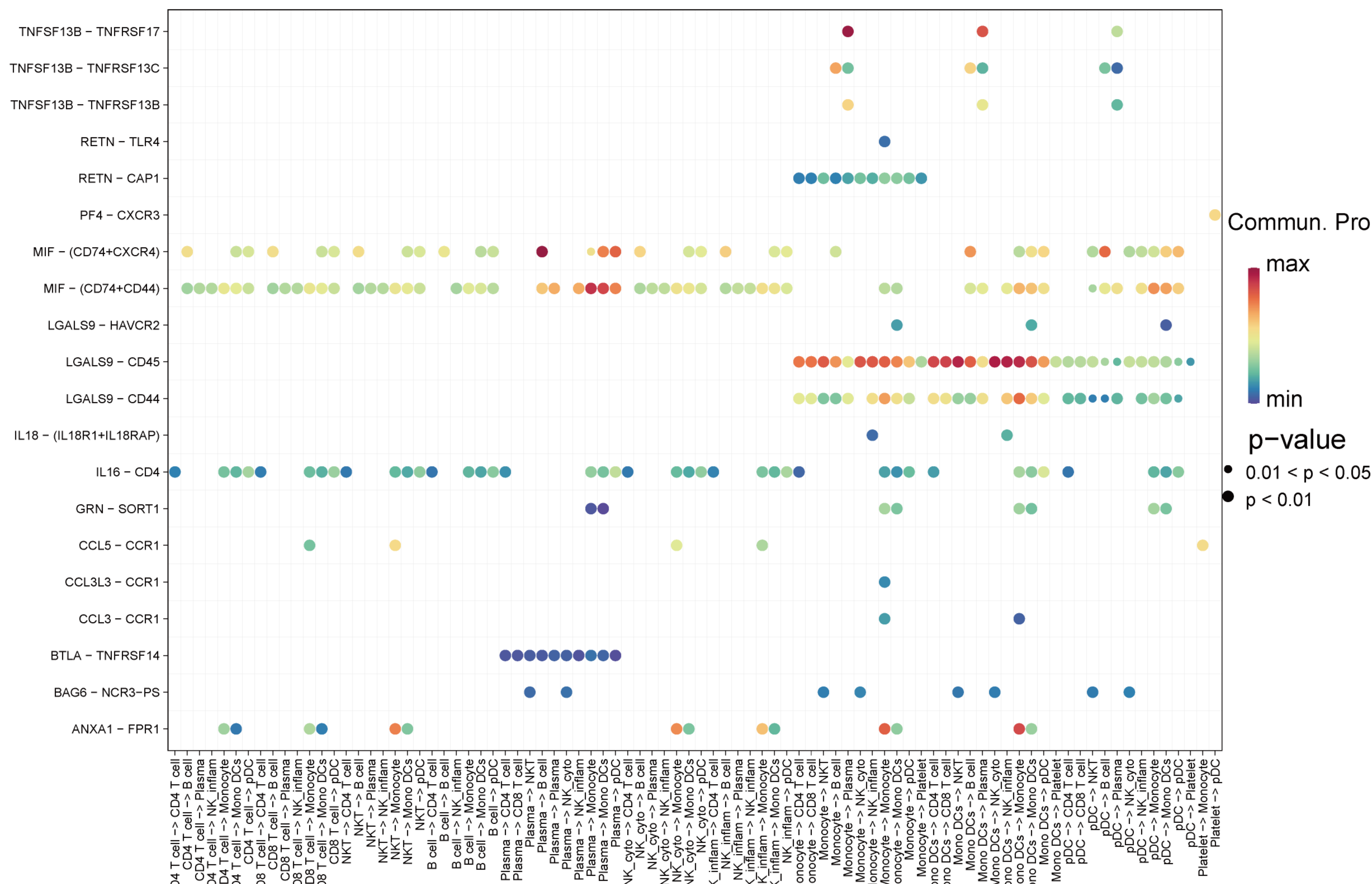

D

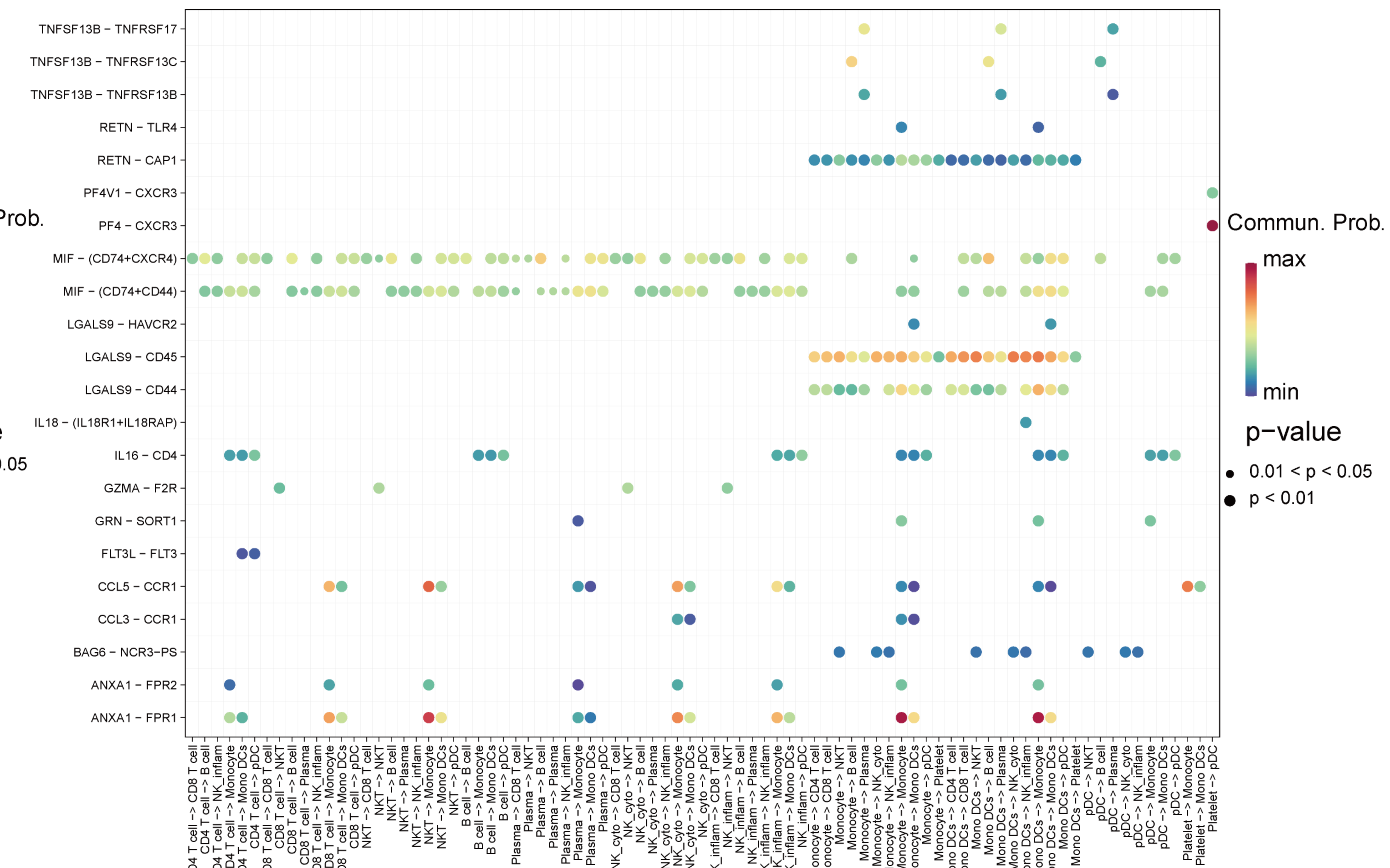

E

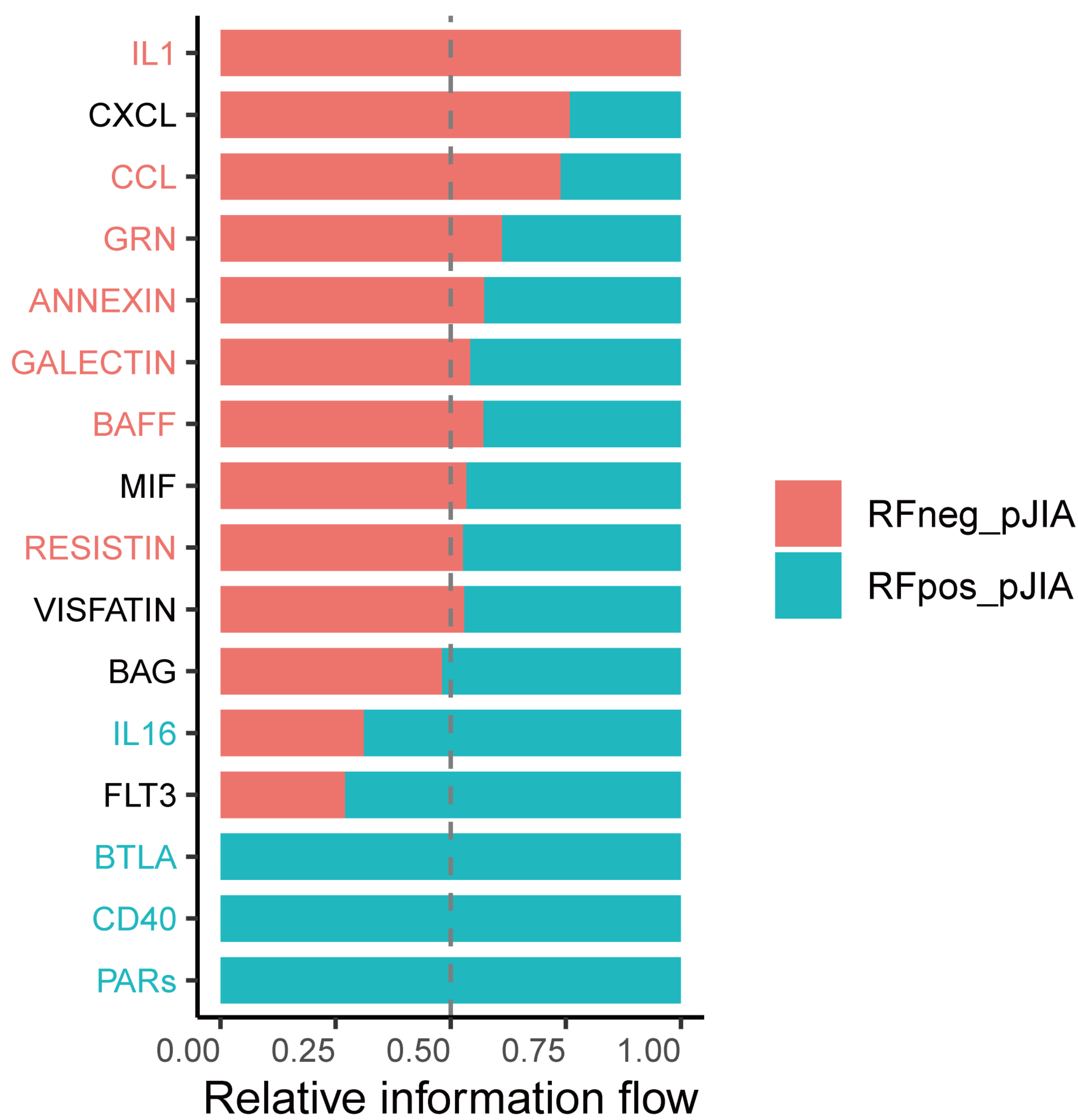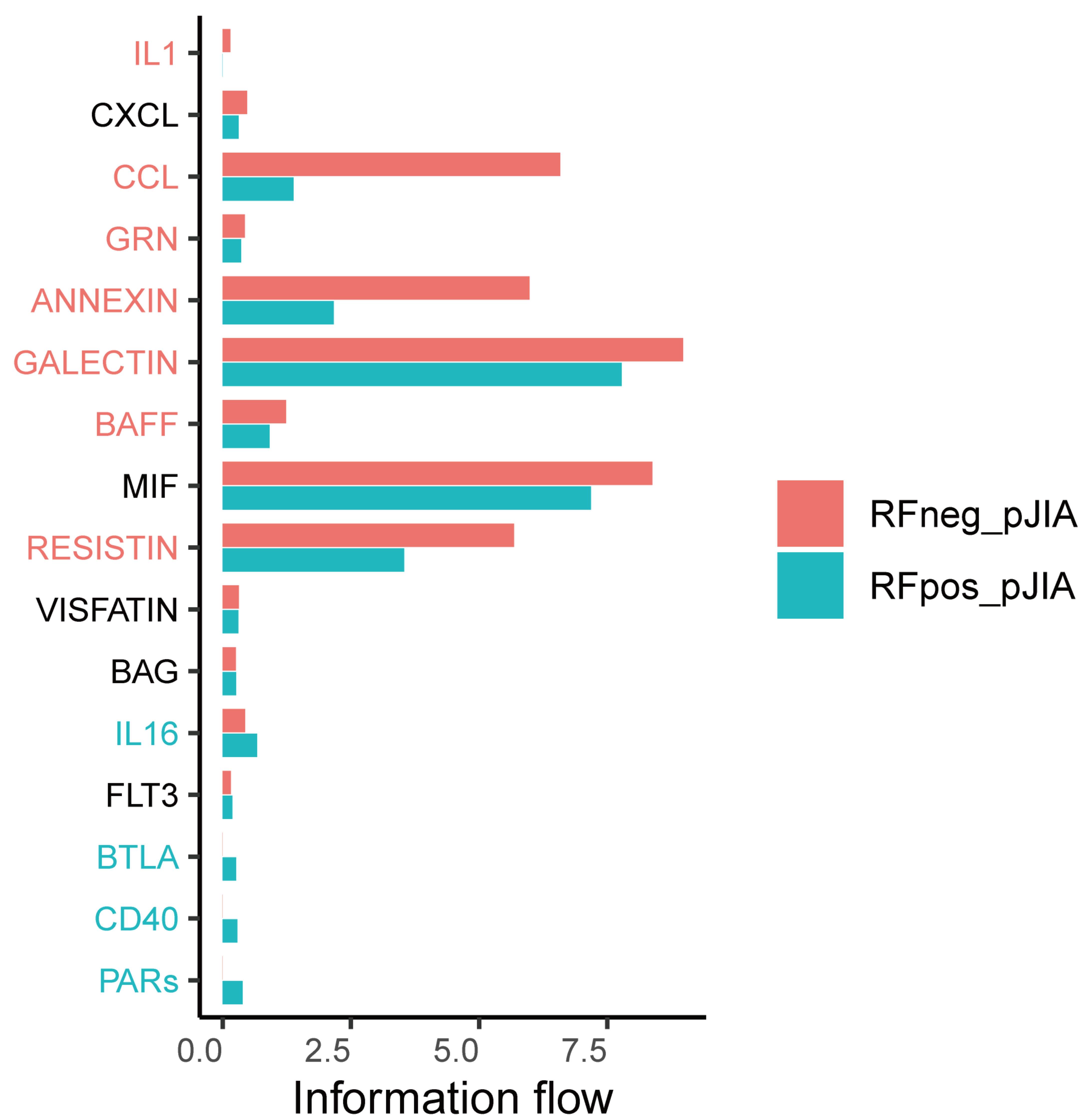

Supplement: Multimedia component 3 — Figure S2 Cellular communication between immune cells. (A–D) Several receptor-ligand pairs interacted between immune cells as predicted by CellChat in RF+ pJIA (A), RF− pJIA (B), oJIA (C), and ERA (D). (E, F) Relative information flow for all pathways and information flow in RF+ pJIA (E) and RF− pJIA (F). RF+ pJIA, rheumatoid factor-positive polyarthritis; RF− pJIA, rheumatoid factor-negative polyarthritis; oJIA, oligoarthritis; ERA enthesitis-related arthritis. [file mmc3.pdf]
