# Supplementary material for: Single-cell landscape of immunological responses in patients with juvenile idiopathic arthritis
Source: Genes Dis. 2025 Mar 3;12(5):101577. doi: 10.1016/j.gendis.2025.101577 (PMC12221592; doi:10.1016/j.gendis.2025.101577)
Supplement: Multimedia component 2 — Figure S1 Single-cell transcriptomic profiles in PBMCs from JIA patients and HCs. (A) Clustering of cell types according to markers, shown by UMAP. Colors indicate expression levels. (B–H) Gene ontology enrichment analysis of DEGs in HC and JIA in CD4+ T cells (B), CD8+ T cells (C), B cells (D), monocytes (E), NK cytotoxicity cells (F), NK inflammation cells (G), and NK T cells (H). PBMCs, peripheral blood mononuclear cells; DEGs, differentially expressed genes; HC, healthy control; NK, natural killer. [file mmc2.pdf]

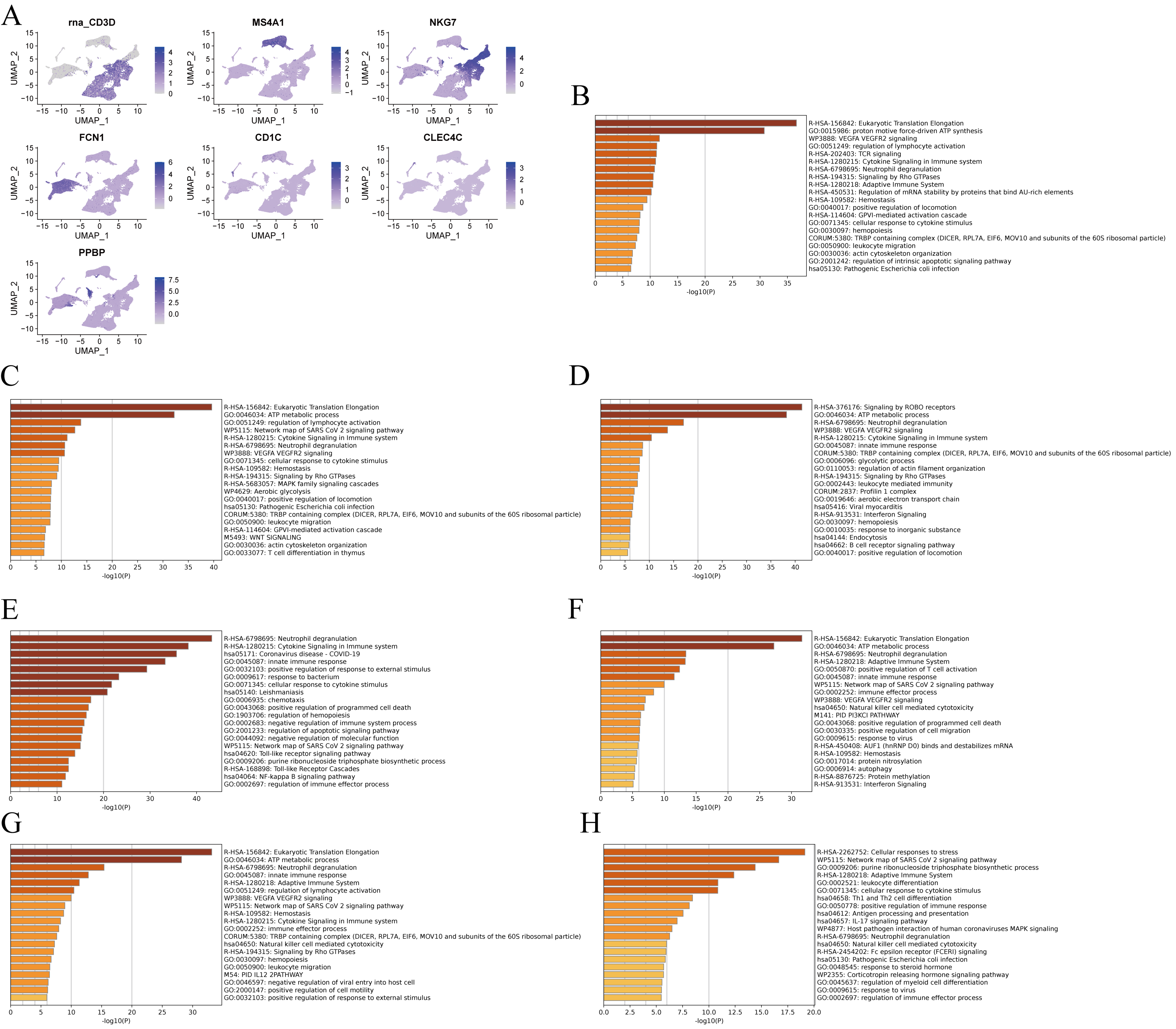

A
rna_CD3D
MS4A1
NKG7
FCN1
CD1C
CLEC4C
PPBP
UMAP_1
UMAP_2
B
R-HSA-156842: Eukaryotic Translation Elongation
GO:0015986: proton motive force-driven ATP synthesis
WP3888: VEGFA VEGFR2 signaling
GO:0051249: regulation of lymphocyte activation
R-HSA-202403: TCR signaling
R-HSA-1280215: Cytokine Signaling in Immune system
R-HSA-6798695: Neutrophil degranulation
R-HSA-194315: Signaling by Rho GTPases
R-HSA-1280218: Adaptive Immune System
R-HSA-450531: Regulation of mRNA stability by proteins that bind AU-rich elements
R-HSA-109582: Hemostasis
GO:0040017: positive regulation of locomotion
R-HSA-114604: GPVI-mediated activation cascade
GO:0071345: cellular response to cytokine stimulus
GO:0030097: hemopoiesis
CORUM:5380: TRBP containing complex (DICER, RPL7A, EIF6, MOV10 and subunits of the 60S ribosomal particle)
GO:0050900: leukocyte migration
GO:0030036: actin cytoskeleton organization
GO:2001242: regulation of intrinsic apoptotic signaling pathway
hsa05130: Pathogenic Escherichia coli infection
-log10(P)
C
R-HSA-156842: Eukaryotic Translation Elongation
GO:0046034: ATP metabolic process
GO:0051249: regulation of lymphocyte activation
WP5115: Network map of SARS CoV 2 signaling pathway
R-HSA-1280215: Cytokine Signaling in Immune system
R-HSA-6798695: Neutrophil degranulation
WP3888: VEGFA VEGFR2 signaling
GO:0071345: cellular response to cytokine stimulus
R-HSA-109582: Hemostasis
R-HSA-194315: Signaling by Rho GTPases
R-HSA-5683057: MAPK family signaling cascades
WP4629: Aerobic glycolysis
GO:0040017: positive regulation of locomotion
hsa05130: Pathogenic Escherichia coli infection
CORUM:5380: TRBP containing complex (DICER, RPL7A, EIF6, MOV10 and subunits of the 60S ribosomal particle)
GO:0050900: leukocyte migration
R-HSA-114604: GPVI-mediated activation cascade
M5493: WNT SIGNALING
GO:0030036: actin cytoskeleton organization
GO:0033077: T cell differentiation in thymus
-log10(P)
D
R-HSA-376176: Signaling by ROBO receptors
GO:0046034: ATP metabolic process
R-HSA-6798695: Neutrophil degranulation
WP3888: VEGFA VEGFR2 signaling
R-HSA-1280215: Cytokine Signaling in Immune system
GO:0045087: innate immune response
CORUM:5380: TRBP containing complex (DICER, RPL7A, EIF6, MOV10 and subunits of the 60S ribosomal particle)
GO:0006096: glycolytic process
GO:0110053: regulation of actin filament organization
R-HSA-194315: Signaling by Rho GTPases
GO:0002443: leukocyte mediated immunity
CORUM:2837: Profilin 1 complex
GO:0019646: aerobic electron transport chain
hsa05416: Viral myocarditis
R-HSA-913531: Interferon Signaling
GO:0030097: hemopoiesis
GO:0010035: response to inorganic substance
hsa04144: Endocytosis
hsa04662: B cell receptor signaling pathway
GO:0040017: positive regulation of locomotion
-log10(P)
E
R-HSA-6798695: Neutrophil degranulation
R-HSA-1280215: Cytokine Signaling in Immune system
hsa05171: Coronavirus disease - COVID-19
GO:0045087: innate immune response
GO:0032103: positive regulation of response to external stimulus
GO:0009617: response to bacterium
GO:0071345: cellular response to cytokine stimulus
hsa05140: Leishmaniasis
GO:0006935: chemotaxis
GO:0043068: positive regulation of programmed cell death
GO:1903706: regulation of hemopoiesis
GO:0002683: negative regulation of immune system process
GO:2001233: regulation of apoptotic signaling pathway
GO:0044092: negative regulation of molecular function
WP5115: Network map of SARS CoV 2 signaling pathway
hsa04620: Toll-like receptor signaling pathway
GO:0009206: purine ribonucleoside triphosphate biosynthetic process
R-HSA-168898: Toll-like Receptor Cascades
hsa04064: NF-kappa B signaling pathway
GO:0002697: regulation of immune effector process
-log10(P)
F
R-HSA-156842: Eukaryotic Translation Elongation
GO:0046034: ATP metabolic process
R-HSA-6798695: Neutrophil degranulation
R-HSA-1280218: Adaptive Immune System
GO:0050870: positive regulation of T cell activation
GO:0045087: innate immune response
WP5115: Network map of SARS CoV 2 signaling pathway
GO:0002252: immune effector process
WP3888: VEGFA VEGFR2 signaling
hsa04650: Natural killer cell mediated cytotoxicity
M141: PID PI3KCI PATHWAY
GO:0043068: positive regulation of programmed cell death
GO:0030335: positive regulation of cell migration
GO:0009615: response to virus
R-HSA-450408: AUF1 (hnRNP D0) binds and destabilizes mRNA
R-HSA-109582: Hemostasis
GO:0017014: protein nitrosylation
GO:0006914: autophagy
R-HSA-8876725: Protein methylation
R-HSA-913531: Interferon Signaling
-log10(P)
G
R-HSA-156842: Eukaryotic Translation Elongation
GO:0046034: ATP metabolic process
R-HSA-6798695: Neutrophil degranulation
GO:0045087: innate immune response
R-HSA-1280218: Adaptive Immune System
GO:0051249: regulation of lymphocyte activation
WP3888: VEGFA VEGFR2 signaling
WP5115: Network map of SARS CoV 2 signaling pathway
R-HSA-109582: Hemostasis
R-HSA-1280215: Cytokine Signaling in Immune system
GO:0002252: immune effector process
CORUM:5380: TRBP containing complex (DICER, RPL7A, EIF6, MOV10 and subunits of the 60S ribosomal particle)
hsa04650: Natural killer cell mediated cytotoxicity
R-HSA-194315: Signaling by Rho GTPases
GO:0030097: hemopoiesis
GO:0050900: leukocyte migration
M54: PID IL12 2PATHWAY
GO:0046597: negative regulation of viral entry into host cell
GO:2000147: positive regulation of cell motility
GO:0032103: positive regulation of response to external stimulus
-log10(P)
H
R-HSA-2262752: Cellular responses to stress
WP5115: Network map of SARS CoV 2 signaling pathway
GO:0009206: purine ribonucleoside triphosphate biosynthetic process
R-HSA-1280218: Adaptive Immune System
GO:0002521: leukocyte differentiation
GO:0071345: cellular response to cytokine stimulus
hsa04658: Th1 and Th2 cell differentiation
GO:0050778: positive regulation of immune response
hsa04612: Antigen processing and presentation
hsa04657: IL-17 signaling pathway
WP4877: Host pathogen interaction of human coronaviruses MAPK signaling
R-HSA-6798695: Neutrophil degranulation
hsa04650: Natural killer cell mediated cytotoxicity
R-HSA-2454202: Fc epsilon receptor (FCERI) signaling
hsa05130: Pathogenic Escherichia coli infection
GO:0048545: response to steroid hormone
WP2355: Corticotropin releasing hormone signaling pathway
GO:0045637: regulation of myeloid cell differentiation
GO:0009615: response to virus
GO:0002697: regulation of immune effector process
-log10(P)
